# Visual motion detection thresholds can be reliably measured during walking and standing

**DOI:** 10.3389/fnhum.2023.1239071

**Published:** 2023-11-09

**Authors:** Stephen DiBianca, John Jeka, Hendrik Reimann

**Affiliations:** Coordination of Balance and Posture, Kinesiology and Applied Physiology, Biomechanics and Movement Science, University of Delaware, Newark, DE, United States

**Keywords:** vision, walking, standing, visual motion detection, psychophysics, sensory threshold

## Abstract

**Introduction:**

In upright standing and walking, the motion of the body relative to the environment is estimated from a combination of visual, vestibular, and somatosensory cues. Associations between vestibular or somatosensory impairments and balance problems are well established, but less is known whether visual motion detection thresholds affect upright balance control. Typically, visual motion threshold values are measured while sitting, with the head fixated to eliminate self-motion. In this study we investigated whether visual motion detection thresholds: (1) can be reliably measured during standing and walking in the presence of natural self-motion; and (2) differ during standing and walking.

**Methods:**

Twenty-nine subjects stood on and walked on a self-paced, instrumented treadmill inside a virtual visual environment projected on a large dome. Participants performed a two-alternative forced choice experiment in which they discriminated between a counterclockwise (“left”) and clockwise (“right”) rotation of a visual scene. A 6-down 1-up adaptive staircase algorithm was implemented to change the amplitude of the rotation. A psychometric fit to the participants’ binary responses provided an estimate for the detection threshold.

**Results:**

We found strong correlations between the repeated measurements in both the walking (*R* = 0.84, *p* < 0.001) and the standing condition (*R* = 0.73, *p* < 0.001) as well as good agreement between the repeated measures with Bland–Altman plots. Average thresholds during walking (mean = 1.04°, SD = 0.43°) were significantly higher than during standing (mean = 0.73°, SD = 0.47°).

**Conclusion:**

Visual motion detection thresholds can be reliably measured during both walking and standing, and thresholds are higher during walking.

## Introduction

Vision plays an important role in balance control for standing and walking by providing information about movement relative to the environment via optical flow (Gibson, 1958). Quantifying the capabilities of the human visual system is challenging, as any particular property such as contrast sensitivity (Owsley, 2003), depth perception (Walk and Gibson, 1961; Brenner and Smeets, 2018), and motion detection (Borst and Egelhaaf, 1989) may affect different functional behaviors. The ability to detect self-motion from optical flow is expected to be most relevant for balance control, but visual motion detection thresholds are typically measured during sitting (Warren et al., 1989; Gilmore et al., 1992; Turano and Wong, 1992; Habak and Faubert, 2000; Freeman et al., 2006, 2008; Conlon et al., 2017), where self-motion is eliminated, and balance is not an issue. Our motivation for this study was to investigate whether: (1) visual motion thresholds can be reliably measured during standing and walking; and (2) to determine whether thresholds differ during balance tasks when self-motion is not constrained.

When studies investigate the relationship between visual processing and fall risk, they typically assess qualities of visual acuity such as contrast sensitivity (Lord and Fitzpatrick, 2001; Wood et al., 2011), depth perception (Felson et al., 1989; Lord and Dayhew, 2001), or size of the visual field (Ivers et al., 1998; Broman et al., 2004). Visual acuity is meaningful for maneuvering around an environment and avoiding falls caused by tripping or hitting obstacles (Broman et al., 2004) as vision provides information about object size, location, and where to place the swing leg foot into a safe space. Visual acuity relates to central vision, or focal vision, capable of high spatial resolution and particularly useful for pattern and object recognition (Larson and Loschky, 2009). While visual acuity mostly concerns central vision, visual motion perception is more related to peripheral vision (Monaco et al., 2007). Illusion of self-motion in response to visual motion, “vection,” has been shown to be primarily influenced by stimuli in the peripheral visual field (Brandt et al., 1973; Tarita-Nistor et al., 2008, 2014). Optic flow can produce illusions of self-motion, and thus disturb upright balance in both standing (Peterka, 2002; Kiemel et al., 2006; Jeka et al., 2010) and walking (Logan et al., 2010; McAndrew et al., 2010; Franz et al., 2015; Reimann et al., 2018). To our knowledge, there has been only one study that has directly compared measures of visual acuity to motion perception in their relationship to control of upright balance. Data collected during the Salisbury Eye Evaluation (SEE Project) (Freeman et al., 2008) found that in a model including visual acuity, contrast sensitivity, visual field, and motion detection threshold, the motion detection thresholds were associated with over three times higher odds of failing on a single leg balance stance task when adjusted for age, sex, and race compared to the other measures of vision. A review by Saftari and Kwon (2018) highlights the general finding that decreased visual acuity is associated with increased risk for falls and hip fractures. Despite these findings, they emphasize that visual motion perception as a contributor to fall risk has been a critical omission in the literature.

Here we tested the reliability of measuring a visual motion detection threshold for detecting optic flow during standing and walking, tasks typically performed for investigating upright balance control. To our knowledge, visual motion detection tests have only been performed while sitting in which the head is typically immobilized (Warren et al., 1989; Gilmore et al., 1992; Turano and Wong, 1992; Habak and Faubert, 2000; Freeman et al., 2006, 2008; Conlon et al., 2017). Here we measured visual motion detection thresholds during standing and walking, where body sway generates a natural background level of self-motion. A threshold measure characterizes the underlying mechanism (Kingdom and Prins, 2009) of a sensory system. In the case of a visual motion detection test, this threshold provides a measure of how sensitive the visual system is in detecting movement in the environment. To calculate a threshold value, perceptual responses are recorded after exposing participants to optic flow stimuli with varying directions and maximum amplitudes. In this study, we use a common adaptive psychophysical method, in which the amplitude of the stimulus is increased or decreased depending on the history of responses. Our hypotheses are that (1) visual motion detection thresholds are correlated between repeated measures in both standing and walking and (2) thresholds in walking are different than in standing.

## Materials and methods

Twenty-nine healthy participants (14 female, 39 ± 15 years old) between the ages of 23 and 67 were recruited for this experiment. Subjects provided informed verbal and written consent to participate. Subjects did not have a history of neurological disorders or visual diagnoses, and no history of surgical procedures involving the legs, spine, or head within 6 months of the protocol. Participants presented with normal or corrected to normal vision (glasses/contacts). The experiment was approved by the University of Delaware Institutional Review Board.

### Experimental protocol and setup

**FIGURE 1:**
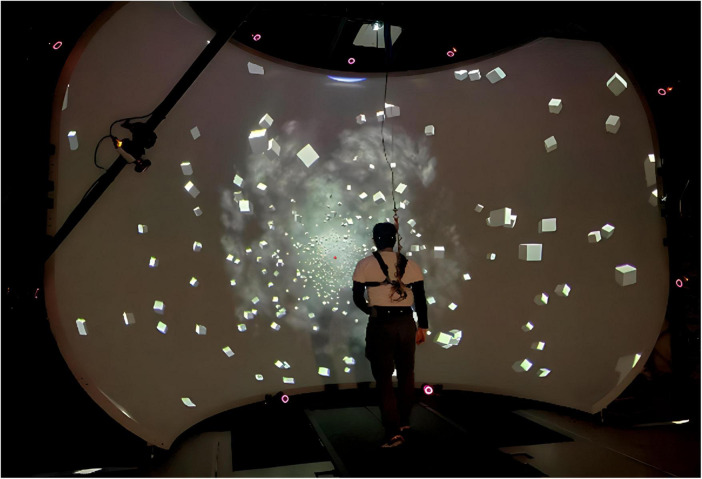
Experimental setup depicting a participant walking in front of the virtual reality dome on the self-paced treadmill.

Participants stood and walked on a self-paced, tied-belt treadmill (Bertec, Columbus, OH, USA) surrounded by a virtual environment displayed on a large dome that occupied the subjects’ full visual field (Figure 1). All participants started with a 15-min walking block to familiarize themselves with the environment, walking on the self-paced treadmill, and the two-alternative forced choice task (2AFC). Ten trials of the 2AFC task were performed at this time. After the familiarization block, participants performed four blocks of the 2AFC task, alternating between standing and walking, with the order counter-balanced between participants, with 15 participants walking first and 14 standing first. In the standing blocks, participants stood 2 m away from the center of the curved screen. Six reflective markers were placed on the subjects: two on the temples, two over the occipital condyles, and two on the posterior superior iliac spines. Marker positions were recorded using a Qualisys Motion Tracker System with 13 cameras at a sampling rate of 200 Hz. For self-paced control of the treadmill, a nonlinear proportional-derivative (PD) controller was implemented via Labview (National instruments Inc., Austin, TX, USA) to keep the midpoint of the two markers on the posterior iliac spine at the midline of the treadmill. With a PD controller, the treadmill speeds up and slows down with the speed of the subject to maintain the subject in the center of the treadmill. Visual perspective and position in the virtual world were linked to the midpoint between the two markers placed on the subjects’ temples and superimposed over the forward motion dictated by the treadmill speed. Subjects wore a safety harness in the event of a fall, although none occurred.

### The virtual scene

The experimental setup is displayed in Figure 1, showing a participant walking on the self-paced treadmill in the virtual environment designed and implemented in Unity3d (Unity Technologies, San Francisco, CA, USA.). The scene consisted of 1,000 cubes floating before a dark background, randomly distributed in a cylindrical tunnel along the anterior-posterior axis with a radius of 14–40 m from the central axis through the treadmill. Each cube was 1 × 1 × 1 m in size. A red sphere was linked to the midpoint between the two markers placed on the temples as a focal point for participants and was placed 50 m ahead in the virtual environment. Fog was displayed in the distance to obfuscate the end of the tunnel and create the perception of infinite distance. The anterior-posterior movement of the virtual scene matched the speed of the treadmill.

### Two-alternative forced choice task

The 2AFC task presents participants with a rotation of the virtual environment around the anterior-posterior axis of the treadmill in a counter-clockwise (left) or clockwise motion (right). Participants were instructed to use the red dot as a focal point and that the cubes would rotate either counter-clockwise, “left,” or clockwise, “right,” around the red dot and to verbally report the direction of motion as “left” or “right.” The stimulus waveform was a single cycle of a raised cosine for velocity with a frequency of 1 Hz. A variable amplitude was determined by the adaptive staircase algorithm such that the screen rotated by the designated amplitude (degrees) per 1 s (see details below). The stimulus was manually triggered by the experimenter at an arbitrary time every 1–2 s after each response. A monotone sound was played during the stimulus in which participants verbally reported the direction of motion as “left” or “right” once the tone ended. This methodology was used for both the standing and walking condition. Each block consisted of 100 trials, where one trial is a single stimulus. After each response, the experimenter initiated the next trial. After every 25 trials the subject was given a brief break to release concentration on the task, then indicated when ready to continue, which typically took about 15 s. In the walking trials, subjects kept walking normally during these breaks. After each block of 100 trials, subjects took longer breaks of at least 2 min, more if needed.

### Adaptive staircase for stimulus amplitude

**FIGURE 2:**
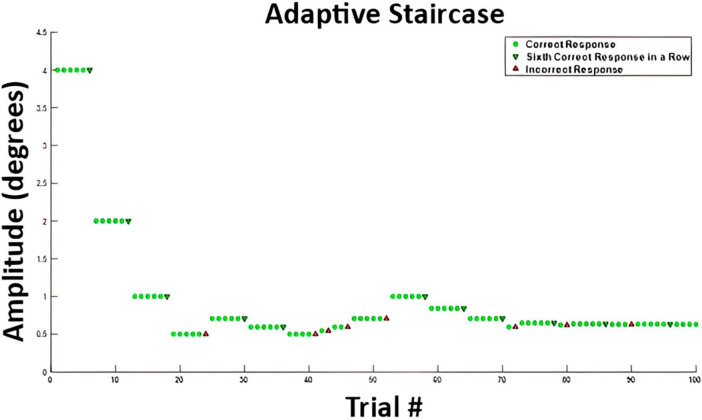
Example stimulus amplitudes from participant VMD04 performing the two-alternative forced choice task while walking. The green circles represent correct answers after which the stimulus amplitude stayed the same. A green downward arrow represents a sixth correct response in a row, which leads to a decrease in stimulus amplitude, making the task more difficult. An upward red arrow represents an incorrect response, which leads to an increase in movement amplitude, making the task easier. This is a 6-down 1-up adaptive staircase.

The amplitude of the stimulus was the maximum angle of rotation around the anterior-posterior axis presented as degrees. The stimulus was generated by the equation:


$$A\times \left(t-\frac{\sin\left(t\times 2\,\pi \times f\right)}{2\,\pi \times f}\right)$$

where *A* is the amplitude of the stimulus (degree), *t* is the time vector, and *f* is the frequency, or 1. To clarify, we define the stimulus in terms of maximum angular amplitude, which co-varies with changes in the velocity of the stimulus. Therefore, an increase in the amplitude would result in an increase in stimulus velocity, and vice versa. These values can also be expressed as peak velocities by taking the first derivative of the stimulus waveform, or, by simply multiplying by a factor of 2. For example, an amplitude of 4° could be expressed as a peak velocity of 8 degree/s. The amplitude was adjusted using an adaptive 6-down 1-up staircase algorithm (Karmali et al., 2016) for parameter estimation by sequential testing (Taylor and Creelman, 1967). A 6-down 1-up adaptive staircase algorithm was implemented based off work by Karmali et al. (2016) who showed a 6-down 1-up staircase provided experimental threshold values closer to theoretical values via Monte Carlo simulations over the more common 3-down 1-up staircase algorithm. The amplitude decreased after six correct responses and increased after one incorrect response, until 100 trials were completed. Figure 2 shows an example from subject VMD04 during their second walking trial of the adaptive staircase protocol. The initial amplitude was always set to 4°.

### Psychometric fit

**FIGURE 3:**
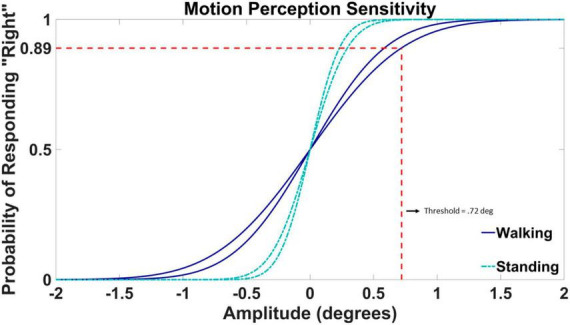
Example psychometric fit data from participant VMD04. Standing fits are represented by cyan dash-dotted lines and walking fits are represented by blue solid lines. The threshold value is highlighted by the dashed red line for walking trial two taken from the staircase data shown in Figure 2. The threshold value is defined by the angle in degrees at which the participant was responding with a rightward rotation with 89% confidence. The amplitude of the rotation is displayed on the horizontal axis, with positive values corresponding to rightward rotations and negative values to leftward rotations. The probability of responding “right” is on the vertical axis.

To obtain a visual motion detection threshold, we fit a psychometric curve to the 100 binary responses of the 2AFC task in each condition. The fit was performed in MATLAB’s fitglm function using a generalized linear model (GLM) with a probit link. The motion detection threshold was defined as the value corresponding to a target probability of $\sqrt[{6}]{0.5}\text{ = 0.89}$ (Taylor and Creelman, 1967; Levitt, 1971; Hall, 1981; Karmali et al., 2016). Figure 3 displays the psychometric fits for participant VMD04. The threshold value is marked on the psychometric fit during walking trial two, the same trial from the adaptive staircase data shown above in Figure 2. The threshold value is highlighted by the dashed red line that corresponds to the amplitude of rotation at which the subject responded “right” with 89% confidence.

The mean and standard deviation of the underlying normal distribution represent the bias and the slope for the psychometric fit. A bias value of 0 indicates an equal chance of left and right guesses (50%) at 0 amplitude movement. The bias of the psychometric curve was set to 0 for all participants. The slope of the psychometric fit is determined by the standard deviation of the underlying Gaussian distribution, which characterizes the acuteness of detection, or how accurate the visual system can detect the stimulus, visual motion (Morgan et al., 2011). Example fits for one participant (VMD04) are shown in Figure 3. Here both the slopes of the walking trials (dark blue) are more shallow than the slopes of the standing trials (cyan), resulting in a larger threshold or a less accurate ability to detect motion in the environment while walking.

### Statistical analysis

The normality and homoscedasticity of the visual motion detection thresholds for both walking and standing per block and per condition were evaluated using a Shapiro–Wilk test of normality and an *F*-test. While the walking threshold data met these assumptions, the standing thresholds were slightly skewed. For Hypothesis 1 on the agreement between threshold measurements obtained in trial one and two, Pearson’s correlation coefficients were calculated between the two measurements of both walking and standing conditions. We used Bland–Altman plots to show the mean difference between the repeated measures and construct limits of agreement (Bland and Altman, 1986). To test for any differences for time and condition on the threshold measures, a two-way repeated measures ANOVA was performed.

## Results

All subjects completed the experiment of both walking and standing conditions. On the individual level, 24 subjects had higher thresholds during walking versus standing. The average walking speed for participants during the walking condition was 1.09 m/s (SD = 0.20 m/s).

### Test-retest reliability

**FIGURE 4:**
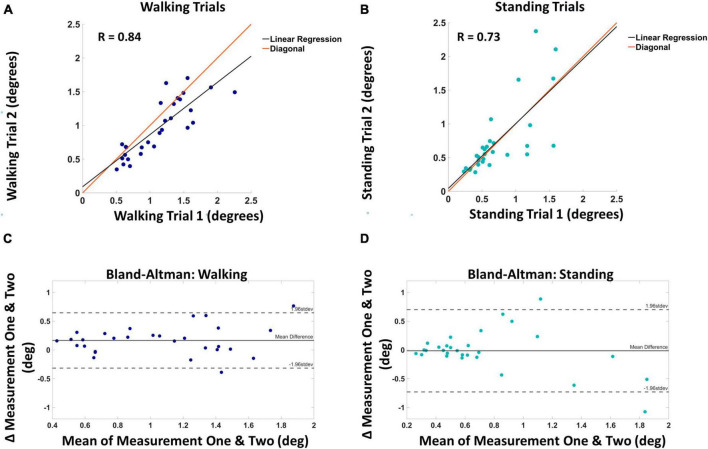
Panel **(A)** shows the visual motion threshold values for walking, with block one on the horizontal and block two on the vertical axis. The red line is the diagonal, and the black line is the linear regression. Panel **(B)** shows the same for standing. Panel **(C)** shows the Bland–Altman plot for walking, with the mean of the two blocks on the horizontal and the difference on the vertical axis. Solid horizontal lines indicate the mean difference and dashed horizontal lines mark two standard deviations from the mean. Panel **(D)** shows the same for standing.

Thresholds were obtained from walking block one (mean = 1.13°, SD = 0.45°), walking two (mean = 0.97°, SD = 0.41°), standing one (mean = 0.73°, SD = 0.41°), and standing two (mean = 0.74°, SD = 0.54°). Both walking and standing conditions showed a strong positive correlation between measurements one and two. The correlation coefficient between the walking measurements was 0.84 (*p* < 0.001), and the correlation coefficient between the standing measurements was 0.73 (*p* < 0.001). Figure 4 displays threshold values from trial one and two against each other for both walking (Figure 4A) and standing (Figure 4B). Also shown in Figure 4 are the Bland–Altman plots for walking (Figure 4C) and standing (Figure 4D). The mean absolute difference between measurement one and two for walking was 0.16° and the mean difference between standing measurements one and two was 0.01°. A two-way repeated measures ANOVA revealed no significant interaction between time and threshold measures *F*(1,1) = 0.792, *p* = 0.375.

We estimated the detection threshold by fitting the slope of the psychometric curve, i.e., the standard deviation of the underlying normal distribution, to the response data for each participant. Bias, or the mean of the underlying normal distribution, can also be used as a parameter for fitting. A bias can occur if a participant either habitually chose a particular side when they were unsure of the direction or if the visual system itself had a skewed mapping (i.e., left movement more noticeable than right). Including the bias can lead to improper fits in rare cases (Fioravanti et al., 2021), and it has been found that participants could voluntarily shift their central bias, causing changes in threshold values, but the slope parameter remained relatively unchanged (Morgan et al., 2011). Since we had no a *priori* reason to expect a bias, we constrained the mean of the psychometric curve to zero for all participants. To investigate possible effects of this choice, we also repeated our analysis with fitting both slope and bias. We found that with this choice, the correlation between the slope estimates decreased for both the walking trials (*R* = 0.57, *p* = 0.001) and standing trials (*R* = 0.67, *p* < 0.001). Additionally, we asked whether the added bias parameter provides meaningful information about the motion perception system for each participant, or rather represents a superfluous parameter that leads to overfitting. To this end, we analyzed the repeatability of the bias estimates between the two trials, using the same approach as for the threshold estimate in the main analysis. We found weak correlation for walking (*R* = 0.35, *p* = 0.064) and standing (*R* = 0.38, *p* = 0.041) as shown in Supplementary Figure 1. Although there was significance for the standing condition, there is little consistency between the bias measures of the two repetitions in both walking and standing, indicating that adding bias as a parameter for the psychometric curve fit represents overfitting rather than meaningful information about the visual system.

### Walking versus standing visual motion thresholds

**FIGURE 5:**
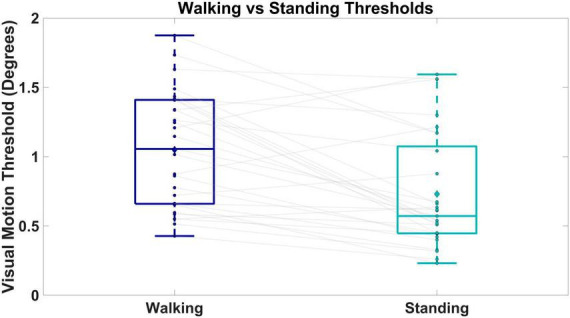
Shows box and whisker plots of visual motion thresholds for walking versus standing. Boxes and whiskers are the median, quartiles, and inter-quartile ranges. Dots are data from individual participants, where each dot is the average between the two repeated measures. Diamonds represent group means. Gray lines connect walking and standing measures from the same participant.

We observed higher visual motion detection thresholds in walking versus standing. Figure 5 shows box and whisker plots of the visual motion detection thresholds for walking (dark blue) and standing (cyan). The two way repeated measures ANOVA revealed a significant effect of condition on the threshold measures [*F*(1,1) = 13.634, *p* < 0.001]. Thresholds were significantly higher during walking compared to standing (*p* < 0.001), with an average threshold value of 1.04° (SD = 0.43) for walking and 0.73° (SD = 0.47) for standing. Twenty-four out of the 29 participants had higher thresholds on average during walking compared to standing.

## Discussion

Our study investigated the reliability of measuring a visual motion detection threshold during walking and standing and compared these measures between those two tasks. We used virtual reality to display a visual scene that rotated in a clockwise or counter-clockwise motion at different amplitudes and asked participants to report the direction of rotation. We found that a visual motion threshold can be reliably obtained during tasks involving body sway and movement, namely standing and walking. We also found evidence that visual motion detection thresholds are higher when walking versus standing, potentially influencing the way visual information is processed between the two tasks.

Visual motion detection thresholds can be reliably obtained during walking and standing. Strong correlations indicate good agreement between the two measures taken at different time points. Bland–Altman plots in Figure 4 support this finding by indicating small average differences between the two measurements for both walking (0.16°) and standing (0.01°). Traditionally, visual motion detection tests are performed while sitting, with the head fixated to avoid any type of head movement (Warren et al., 1989; Gilmore et al., 1992; Turano and Wong, 1992; Wood and Bullimore, 1995; Tran et al., 1998; Habak and Faubert, 2000; Snowden and Kavanagh, 2006; Freeman et al., 2006, 2008; Conlon et al., 2017). These tests have explored different aspects of motion detection and it is unclear which are most relevant for balance. For example, minimal displacement thresholds have been quantified using the translation of a random dot pattern (Habak and Faubert, 2000; Snowden and Kavanagh, 2006; Conlon et al., 2017), motion coherence thresholds have been quantified by translating select percentages of those dots (Gilmore et al., 1992; Tran et al., 1998; Snowden and Kavanagh, 2006), speed discrimination thresholds have been quantified by varying the object motion’s speed (Snowden and Kavanagh, 2006), and heading direction thresholds have been quantified by varying optic flow patterns relevant to a vertical object (Warren and Hannon, 1988; Warren et al., 1989). These studies all implement psychophysical testing, however, there is no standardized approach, using different methods such as the BEST PEST (Gilmore et al., 1992; Habak and Faubert, 2000), method of constant stimuli (Turano and Wong, 1992; Tran et al., 1998), or adaptive staircases (Wood and Bullimore, 1995; Snowden and Kavanagh, 2006; Freeman et al., 2006, 2008). Furthermore, these studies have manipulated and controlled for various characteristics of the stimuli such as luminance and contrast. Again, it is unclear which of these aspects of motion discrimination is most relevant to balance, thus motivating our study to see if motion discrimination is viable to explore during upright balance tasks such as standing and walking with the inherent added head movement. Immobilizing the head eliminates retinal slip caused by natural head sway during standing and walking. This may explain why values of motion discrimination are considerably smaller while sitting (Turano and Wong, 1992; Snowden and Kavanagh, 2006; Freeman et al., 2006, 2008; Conlon et al., 2017), obtaining values ranging from 0.009° to 0.121°. Heading direction thresholds obtained by Warren and Hannon (1988), and Warren et al. (1989) may be more relevant to upright balance control since navigating through our environment via optic flow is important for locomotion. Thresholds calculated via heading direction are similar to what is seen here, ranging from 1.1° to 1.9°. Although it must be noted that motion thresholds here cannot be directly compared to past literature due to methodological differences such as psychophysical protocols, and stimulus conditions such as luminance, contrast, and type of motion. Here, we used a rotation of the virtual scene to measure motion detection. This type of stimulus was selected based on previous work investigating the role of vision and balance during walking in which visual perturbations have been implemented around the anterior/posterior axis to simulate the sensation of a fall and investigate medial/lateral balance control (McAndrew et al., 2010; Franz et al., 2015; Reimann et al., 2018).

The visual motion stimulus presented here is in terms of maximum angular amplitude per 1 s, resulting in changes of both displacement and velocity of the stimulus during the adaptive staircase protocol to quantify a visual motion threshold. Visual motion processing has been generally viewed as having two distinct processes: first and second order processing. First order processing is defined by differences in luminance while second order processing is defined by differences in contrast, texture, or depth (Seiffert and Cavanagh, 1998; Habak and Faubert, 2000; Baker and Mareschal, 2001). Seiffert and Cavanagh (1998) have shown that second order processing is position based and support findings from Nakayama and Tyler (1981) that first order processing is velocity based. Since both speed and amplitude were manipulated in this experiment, we expect that a combination of first order and second order visual processing were used to detect the movement of the stimuli. It is still undetermined as to which parameter is more or less useful for controlling upright balance. To note, it has been suggested that the loss of accurate velocity estimations from a sensory modality based on modeling work from Kiemel et al. (2002) are more problematic to upright postural control than position estimates (Jeka et al., 2004). Snowden and Kavanagh (2006) provide evidence that speed discrimination is hindered in older adults compared to healthy young. The loss of velocity dependent information in visual motion processing could potentially be an underlying mechanism to hindered upright balance control. Since the stimulus presented here contains both a position and speed component, the underlying neural mechanism cannot be attributed to one or the other.

Our motivation was to perform the psychometric tests in an ergonomic manner that would quantify visual motion thresholds during natural movements of the head and body while maintaining upright balance, as opposed to a restrained position. Since participants were not restrained in any way, potential cues from the vestibular and proprioceptive system from the natural motion may have influenced the participants’ threshold results. Although small, a visual stimulus can provoke the illusion of a fall and cause a balance response that may cause added movement of the head and/or ankles, cueing the vestibular or proprioceptive system (Reimann et al., 2018). Some subjects reported perception of self-motion when the visual stimulus became small, rather than movement of the visual stimulus. Such cues from other sensory systems may have influenced responses that could not be considered purely visual. Head movements in standing are relatively small, but walking produces considerable head movement and might influence a person’s ability to detect motion depending on the direction in which the stimulus is moving relative to the head. For the walking conditions, the visual stimulus was manually triggered by the experimenter with a 1–2 s time window between stimuli regardless of the phase of the gait cycle. For example, the visual stimulus may have rotated to the right while the participant was swaying to the left or right, which would add to or reduce visual motion on the retina, respectively. Controlling the onset of the visual stimulus relative to the gait cycle may reduce the variability of thresholds measured in the current investigation.

Beyond accurate estimates of visual detection thresholds, it may be beneficial to understand if the influence of visual movement on upright balance changes during the gait cycle. For example, during double stance, more information is available from lower limb proprioceptors and thus may lead to less reliance on vision. In contrast, during single stance, in which the contralateral leg is in swing phase, visual cues may be more important to maintain balance since there is less contact with the body to the ground. In fact, phase-dependent visual coupling has been observed during walking (Logan et al., 2010). One major mechanism for balance control is modulation of the foot placement based on the state of the body at mid-stance (Wang and Srinivasan, 2014), and visual motion detection is likely used to estimate the body state (i.e., CoM position and velocity).

The influence of age on visual motion thresholds during standing and walking is under studied. Previous literature has indicated an increase in visual motion detection thresholds for older adults compared to young adults (Warren et al., 1989; Gilmore et al., 1992; Tran et al., 1998; Habak and Faubert, 2000; Snowden and Kavanagh, 2006; Conlon et al., 2017), but there are no studies that attempt to measure thresholds in older adults during standing and walking. Older adults are known to place more emphasis on vision (i.e., upweight) while standing (Haibach et al., 2009) and walking (Franz et al., 2015). If motion thresholds are higher for older adults during standing and walking, combined with their higher reliance on vision for upright balance control, larger thresholds may contribute to their fall risk. Saftari and Kwon (2018) emphasize that particularly in the aging literature, the relationship between visual detection thresholds and fall-risk remains a critical knowledge gap.

Overall, our results indicate that a visual motion detection threshold can be reliably measured during walking and standing, and that thresholds are higher for walking than standing. The relationship between visual processing and fall risk has focused on aspects of visual acuity, although not in the case of optic flow detection. The ability to reliably measure a visual motion detection threshold while standing and walking adds to our understanding of visual motion processing and balance control, particularly in populations with higher fall risk.

## Data availability statement

The raw data supporting the conclusions of this article will be made available by the authors, without undue reservation.

## Ethics statement

The studies involving humans were approved by the University of Delaware IRB, Newark, DE. The studies were conducted in accordance with the local legislation and institutional requirements. The participants provided their written informed consent to participate in this study. Written informed consent was obtained from the individual(s) for the publication of any potentially identifiable images or data included in this article.

## Author contributions

SD and HR contributed to the conception, implementation, and analysis of this study. SD ran the study and drafted the original manuscript. HR and JJ reviewed and edited the manuscript for publication. All authors contributed to manuscript revision, read, and approved the submitted version.
