# Supplementary figures and images for: Visual motion detection thresholds can be reliably measured during walking and standing
Source: Front Hum Neurosci. 2023 Nov 9;17:1239071. doi: 10.3389/fnhum.2023.1239071 (PMC10665501; doi:10.3389/fnhum.2023.1239071)

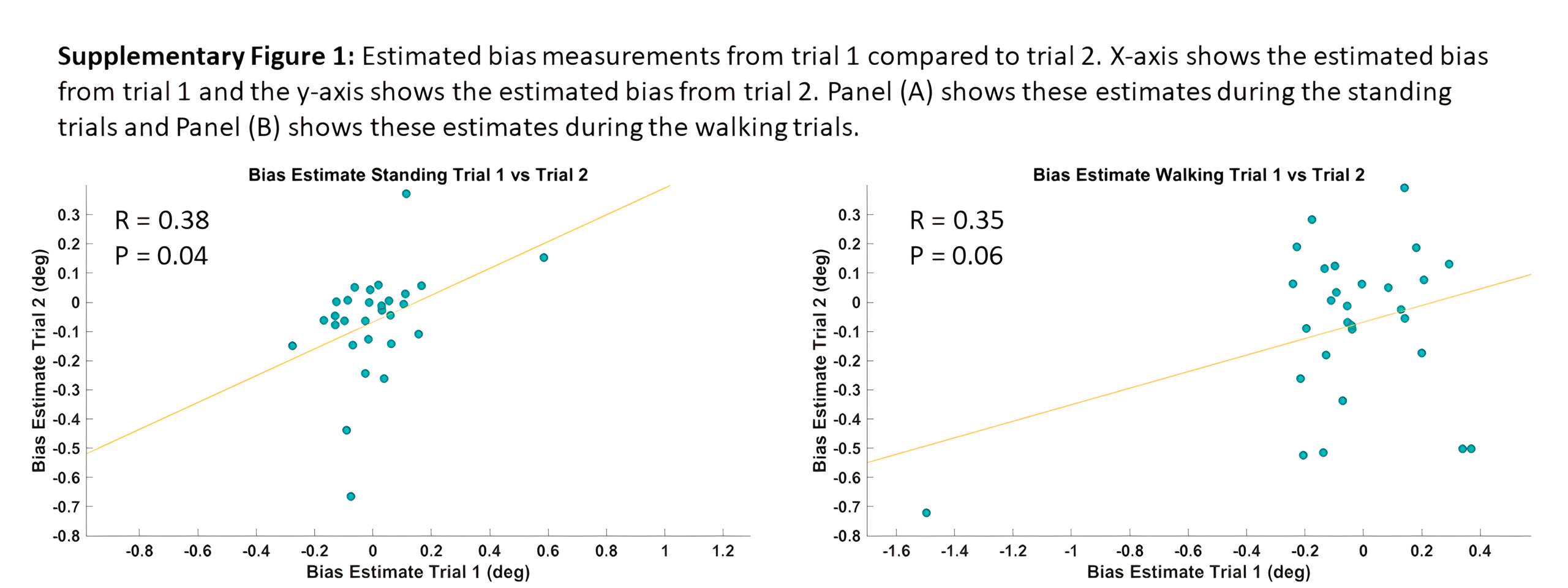

Supplement: Supplementary file 1 [file Image_1.TIFF]
